# A new species of squat lobster of the genus *Hendersonida* (Crustacea, Decapoda, Munididae) from Papua New Guinea

**DOI:** 10.3897/zookeys.935.51931

**Published:** 2020-05-21

**Authors:** Paula C. Rodríguez-Flores, Enrique Macpherson, Annie Machordom

**Affiliations:** 1 Centre d’Estudis Avançats de Blanes (CEAB-CSIC), C. acc. Cala Sant Francesc 14 17300 Blanes, Girona, Spain Centre d’Estudis Avançats de Blanes Girona Spain; 2 Museo Nacional de Ciencias Naturales (MNCN-CSIC), José Gutiérrez Abascal, 2, 28006 Madrid, Spain Museo Nacional de Ciencias Naturales Madrid Spain

**Keywords:** Anomura, mitochondrial genes, morphology, West Pacific

## Abstract

*Hendersonida parvirostris***sp. nov.** is described from Papua New Guinea. The new species can be distinguished from the only other species of the genus, *H. granulata* (Henderson, 1885), by the fewer spines on the dorsal carapace surface, the shape of the rostrum and supraocular spines, the antennal peduncles, and the length of the walking legs. Pairwise genetic distances estimated using the 16S rRNA and COI DNA gene fragments indicated high levels of sequence divergence between the new species and *H. granulata*. Phylogenetic analyses, however, recovered both species as sister species, supporting monophyly of the genus.

## Introduction

Squat lobsters of the family Munididae Ahyong, Baba, Macpherson & Poore, 2010 are recognised by the trispinose or trilobate front, usually composed of a slender rostrum flanked by supraorbital spines (Ahyong et al. 2010; Macpherson and Baba 2011). The family is one of the most diverse of the anomuran decapods, containing 21 genera and more than 400 species distributed in the Atlantic, Indian, and Pacific oceans, from the coastal area to the abyssal plain (Baba et al. 2008; Schnabel et al. 2011). Among these genera, the genus *Paramunida* Baba, 1988 was proposed based on the presence of spinules or granules densely covering the carapace dorsally (rather than transverse ridges), the short undeveloped rostrum, the antennal peduncle with a well-developed anterior prolongation of article 1, and the absence of the first pair of male gonopods (Baba 1988). *Hendersonida* Cabezas & Macpherson, 2014 was proposed for one species, *Paramunida granulata* (Henderson, 1885), widely distributed from the Philippines to Polynesia. *Hendersonida* is closely related to a clade corresponding to the genus *Paramunida* Baba, 1988 (Cabezas et al. 2012; Cabezas and Macpherson 2014). The two genera show genetic divergences values higher than expected between species, and within the range observed between other genera of munidid squat lobsters (Cabezas et al. 2011; Cabezas and Macpherson 2014). They can also be morphologically differentiated by differences in the spinulation of the carapace and the length of the distomesial spine of the antennal article 2 (Cabezas and Macpherson 2014).

During a recent expedition to Papua New Guinea in August-October 2010 (BIOPAPUA) (Macpherson et al. in press), two specimens of a species of squat lobster of *Hendersonida* were collected. The morphological and molecular analyses of these specimens indicated that they differ from the type species of *Hendersonida*. Therefore, the specimens are described and illustrated here as a second species of the genus.

## Materials and methods

### Sampling and identification

Specimens were collected using beam trawls in August-October 2010 (BIOPAPUA) expedition to Papua New Guinea. The types are deposited in the collections of the Muséum national d’Histoire naturelle, Paris (**MNHN**). The terminology employed in the description largely follows Baba et al. (2009) and Macpherson and Baba (2011). The size of the carapace indicates the postorbital carapace length measured along the dorsal midline from the posterior margin of the orbit to the posterior margin of the carapace. The length of each pereopod article is measured in lateral view along its extensor margin (excluding distal spine), the breadth is measured at its widest portion.

### Molecular analysis

Tissue of each specimen was isolated from the muscle of pereopod 5 and homogenised overnight with 20 µl proteinase K in 180 µl of buffer ATL (QIAGEN). The extraction was performed using DNeasy Blood and Tissue Kit following manufacturer instructions (QIAGEN). Two molecular markers were amplified: a fragment from the mitochondrial cytochrome oxidase subunit I (COI) using primers LCO1490 (Folmer et al. 1994) and COI-H (Machordom et al. 2003), and a 16S rRNA (16S) fragment, using 16SAR-16SBR from Palumbi et al. (2002) pair of primers.

The pre-mixing of the PCR reagents was built in 25 µl total volume, which included 2 µl of DNA extracted, 0.2 mM of each deoxyribonucleotide triphosphate (dNTP), 0.2 µM of each primer forward and reverse, 2U of MyTaq polymerase (Bioline), 5 µl of 5× buffer solution with MgCl_2_ and sterilised H_2_O. PCR amplification was performed with a thermal cycle including an initial denaturation of 94–95 °C for 1–4 min and 40 cycles with 95 °C for 1 min, annealing in 42–45 °C for 1 min followed by an extension set on 72 °C for 1 min. A final extension cycle at 72 °C was set for 10 min. The amplicons were visualised in agarose 1% gels and purified using ExoSAP-IT™ PCR Product Cleanup Reagent (Thermo Fischer) before sequencing. The purified products were sent to Secugen S.L. (Madrid) for DNA Sanger sequencing.

The nucleotide sequences of both forward and reverse were visualised and assembled with Sequencher 4.10.1 software package (Gene Codes Corp.). Multiple sequence alignment for the 16S genes was carried out in MAFFT (Katoh et al. 2002) and the revised in AliView (Larsson 2014). Uncorrected-p pairwise distances between species were calculated in PAUP (Swofford 2002), using the sequences from Cabezas et al. (2012) and McCallum et al. (2016). All the obtained sequences were submitted to GenBank. To test the monophyly of *Hendersonida*, we included all genetic data available from *Paramunida* species included in Cabezas et al. (2012) and McCallum et al. (2016). We collapsed the *Paramunida* node to facilitate the comparison between genera. Sequences of *Paramunida* spp., *Hendersonida granulata* and *Agononida indocerta* were obtained from GeneBank.

Bayesian phylogenetic analysis was performed in MrBayes v3. 2. 1 (Huelsenbeck and Ronquist 2001) using a matrix with the concatenated COI and 16S partial genes. *Agononida indocerta* was selected as the outgroup (GenBank accessions: KM281837.1, KM281818.1). The run was performed in CIPRES portal (Miller et al. 2010). To estimate the posterior probabilities, four Markov Chains Monte Carlo (MCMC) were run for 2 × 10^7^ generations sampling trees and parameters every 20000 generations. The initial 25 % of the generations were discarded as burn-in. The phylogenetic tree was visualised and edited in FigTree v1. 4. 2 (Rambaut 2014); nodes posterior probabilities from the Bayesian Inference were included.

## Results

**Figure 1.**
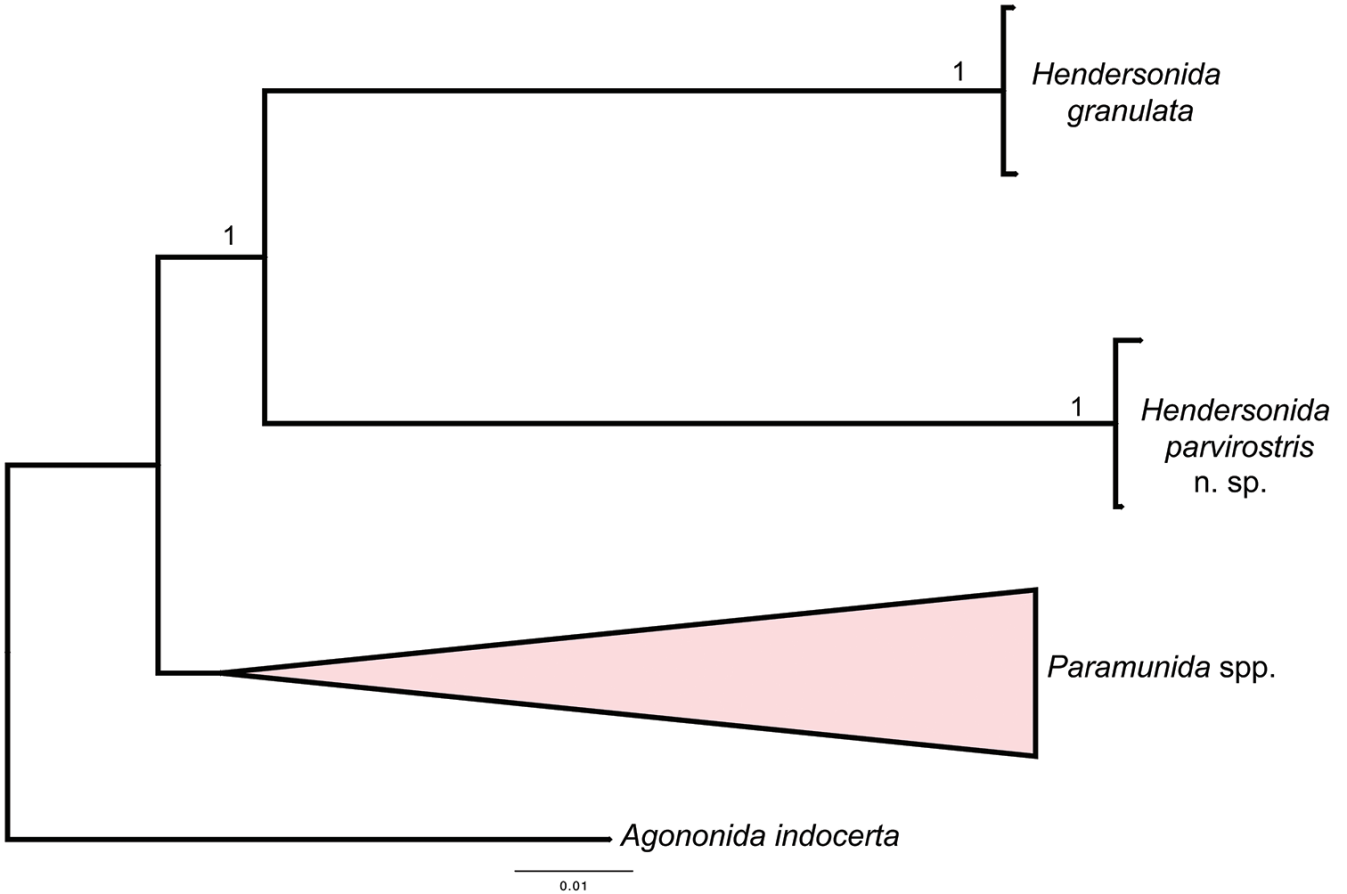
Phylogenetic hypothesis based on mitochondrial molecular data (COI and 16S) represented by a tree obtained by Bayesian inference, including Bayesian posterior probabilities. To test the monophyly of *Hendersonida*, we have included all genetic data available from species of *Paramunida* included in Cabezas et al. (2012) and McCallum et al. (2016) (38 species). We have collapsed the *Paramunida* node to facilitate comparison between genera.

Our results demonstrated the existence of two species of *Hendersonida* supported both by molecular and morphological characters. Both species formed a clade with high Bayesian posterior probability (Fig. 1) and with high genetic distances (11% and 16% for the 16S and COI, respectively). The uncorrected-p distance values between the species of *Hendersonida* and the species of *Paramunida* obtained a range from 9% to 13% for the 16S and from 14% to 19% for the COI.

### Systematic account

**Superfamily Galatheoidea Samouelle, 1819**

**Family Munididae Ahyong, Baba, Macpherson & Poore, 2010**

**Genus *Hendersonida* Cabezas & Macpherson, 2014**

#### Hendersonida parvirostris sp. nov.

Taxon classificationAnimalia

E228A351-A44F-5A05-A40B-83DC73ADDF5A

http://zoobank.org/603BB920-7558-4FA4-BB27-8058768F3668

Figures 2 , 3

##### Material.

***Holotype***: Biopapua stn CP3645, 24/8/2010, 06°46.394'S, 147°50.605'E, 403–418 m: ovigerous female, 8.9 mm (MNHN-IU-2011-4498). ***Paratype***: Biopapua. Stn CP3633, 22/8/2010, 06°51.841'S, 147°04.672'E, 395–406 m: 1 ovigerous female, 12.2 mm (MNHN-IU-2011-3379).

##### Diagnosis.

Rostrum shorter than supraocular spines, each supraocular spine with small lateral spine. Carapace dorsal surface granulated with few scattered minute spines. Thoracic sternites with numerous arcuate striae, sternite 4 narrowly contiguous to sternite 3. Abdominal somites 2 and 3 spinose. Distomesial spine of antennal article 2 reaching end of article 3. Extensor distal margin of maxilliped 3 armed. Pereopods 2–4 long and slender, merus ca. 25 times as long as wide.

##### Description.

***Carapace***: Slightly broader than long; dorsal surface covered with numerous granules and few scattered minute spines, with few short simple setae; epigastric region with row of 6 minute spines; mesogastric region slightly convex, unarmed; cervical groove distinct; cardiac and anterior branchial regions slightly circumscribed; cardiac region with anterior transverse row of four minute spines, and two minute spines posterior to it; each branchial region with 2–4 small spines near lateral borders; frontal margin slightly concave; lateral margins convex; anterolateral spine reaching sinus between rostral and supraocular spines. Rostrum very short; supraocular spines longer than rostrum, each spine with additional small lateral spine; margin between rostral and supraocular spines concave.

***Sternum***: Thoracic sternites with numerous arcuate striae; sternite 3 width less than half width of sternite 4, anterior margin nearly straight; sternite 4 with anterior margin moderately elongate, narrowly contiguous to sternite 3; sternite 7 with numerous granules.

***Abdomen***: Somites 2 and 3 each with some small or moderate–sized spines on anterior and posterior ridges, two median spines larger than others; posterior ridge of somite 4 without distinct single median spine.

***Eyes***: Large, cornea dilated, much wider than eyestalk.

***Antennule***: Article 1 barely exceeding corneae, with distomesial spine slightly shorter than distolateral; ca. twice as long as wide; lateral margin without fringe of long setae, with distal slender portion ca. half as long as proximal inflated portion.

***Antenna***: Anterior prolongation of article 1 overreaching antennular article 1 by ca. one-fourth of its length; article 2 shorter than article 3 and slightly longer than wide, ventral surface with small scales; distomesial spine well developed, reaching end of article 3, and clearly not reaching midlength of anterior prolongation of article 1, distolateral angle unarmed; article 3 twice longer than wide, unarmed.

***Maxilliped* 3**: Ischium 1.5 times length of merus measured along dorsal margin, distoventrally bearing one spine; merus with two or three small spines on flexor margin, extensor margin with distal spine.

***Pereopod* 1**: Lost in holotype, only merus preserved in paratype. Merus 2.5 times carapace length, ca. 15 times longer than high, with row of spines along mesial margin.

***Pereopods* 2–4**: Similar, long and slender, with minute granules and short scales on ventrolateral sides of meri, carpi and propodi; scales with short setae; extensor and flexor margins with numerous long plumose setae; pereopod 2 6.0 times carapace length, merus 3.0 times longer than carapace, ca. 25 times as long as wide, 1.8 times as long as propodus; propodus 20 times as long as wide, and 1.7 times dactylus length; merus with well-developed spines along extensor border, flexor margin with few spines; carpus with distal spine on extensor and flexor margin; propodus with some small movable spines along flexor margin; dactylus slightly curved, with longitudinal carinae along mesial and lateral sides, ventral border, under flexor margin, unarmed; pereopods 3 and 4 of similar length as pereopod 2, with similar spinulation and segment proportions as pereopod 2.

***Colour in life***: Base colour of carapace light orange, gastric region reddish; granules and spines orange. Rostrum and supraocular spines reddish. Abdominal somites 1–4 light orange, with scales and granules orange or reddish; somites 5 and 6 and telson whitish. Pereopods 2–4 light orange, spines along flexor margins reddish, spines along flexor margins whitish; distal portion of meri, carpi, and propodi and proximal part of carpi, propodi, and dactyli with reddish band, distal half of dactyli whitish.

**Figure 2.**
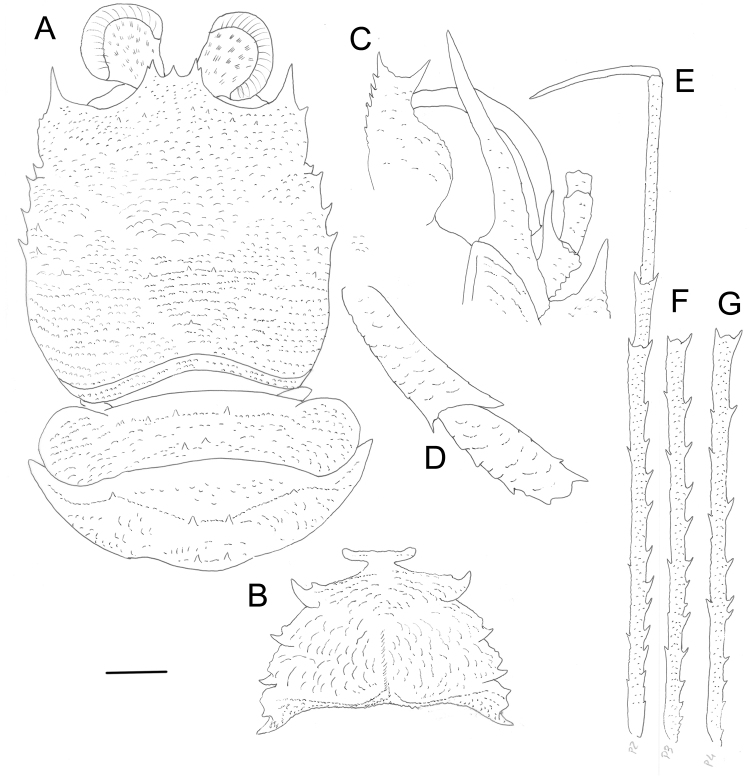
*Hendersonida parvirostris* sp. nov. ovigerous female holotype, 8.9 mm (MNHN-IU-2011-4498), Papua New Guinea. **A** Carapace and abdomen, dorsal view **B** sternum **C** left antennule and antenna, ventral view **D** right maxilliped 3, lateral view **E** left pereopod 2, lateral view **F** left pereopod 3 merus, lateral view **G** left pereopod 4 merus, lateral view. Scale bars: 4 mm (**A, B, E, F, G**); 8 mm (**C, D)**.

##### Genetic data.

GenBank accession numbers: 16S MT252616–MT252617, and COI MT250542–MT250543 (Fig. 1).

##### Etymology.

From the Latin, *parvus*, little, and *rostrum*, in reference to the small size of the rostral spine.

##### Remarks.

The genus *Hendersonida* was erected for one rare species, *H. granulata* (Henderson, 1885) known from several localities of the western Pacific, clearly differentiable from all species of the genus *Paramunida* Baba, 1988 (Macpherson 1993; Cabezas et al. 2010). The new species, *H. parvirostris*, is the second representative of the genus. Both species are morphologically and genetically separated from all the species of *Paramunida* (Fig. 1).

Two conspicuous diagnostic characteristics differentiate the *Hendersonida* genus from *Paramunida*: the granulated surface of the carapace; and the long distomesial spine of antennal article 2, almost reaching the end of the anterior prolongation of article 1 (Cabezas and Macpherson 2014). The morphology of the new species, however, shows that the long distomesial spine of antennal article 2 is not a valid generic character because this spine is moderately short, only reaching the end of antennal article 3, in *H. parvirostris*. Additionally, in both species the anterior margin of sternite 4 is moderately elongate, narrowly contiguous with sternite 3, whereas this margin is nearly transverse, broadly contiguous with sternite 3, in *Paramunida*.

The two species of *Hendersonida* can be differentiated by the following characters:

- The dorsal carapace surface has numerous well-developed spines in H. granulata, whereas these spines are minute and nearly absent in H. parvirostris.
- The supraocular spines slightly exceed the rostral spine in the new species, whereas the rostral spine clearly overreaches the supraocular spines in H. granulata. Furthermore, each supraocular spine has one additional small lateral spine in the new species, which are absent in H. granulata.
- The distomesial spine of the antennal article 2 reaches the end of the article 3 in the new species, whereas this spine almost reaches the end of the anterior prolongation of the article 1 in H. granulata.
- The extensor margin of maxilliped 3 merus has a distal spine in the new species, whereas this spine is absent in H. granulata.
- Pereopods 2–4 are much longer and slender in the new species: propodus 20 times as long as wide in H. parvirostris and 7–8 times as long as wide in H. granulata (Cabezas et al. 2010).

##### Distribution.

Papua New Guinea, between 395 and 418 m.

**Figure 3.**
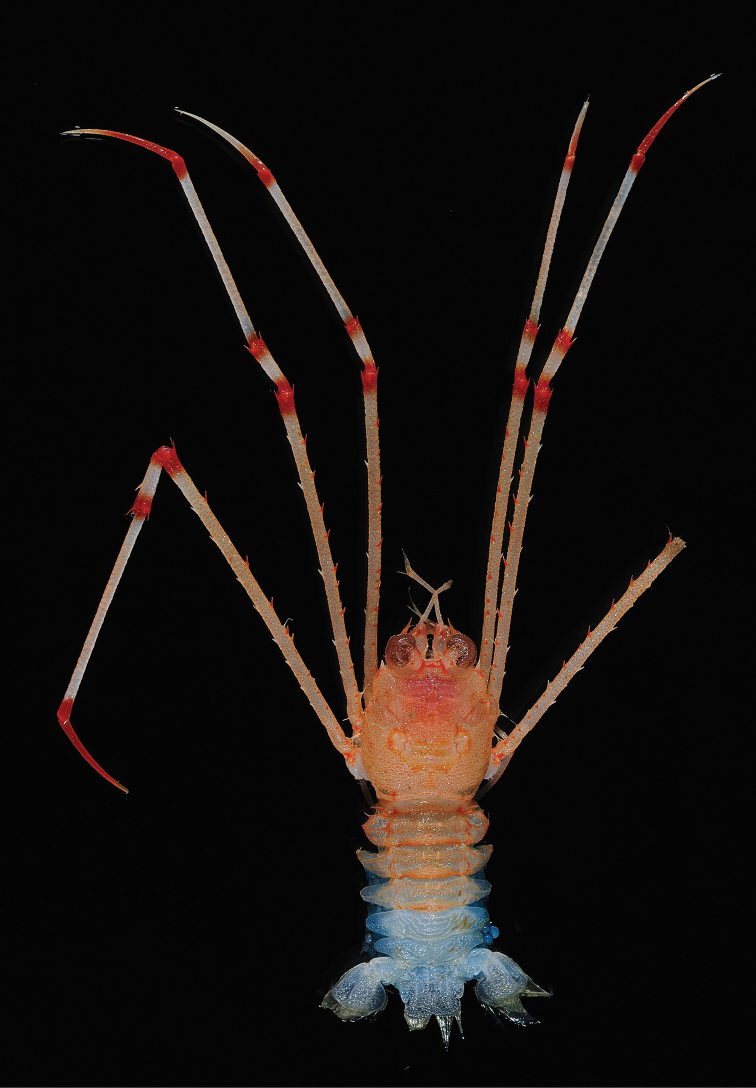
*Hendersonida parvirostris* sp. nov. dorsal view of ovigerous female holotype, 8.9 mm (MNHN-IU-2011-4498), Papua New Guinea.

## Discussion

Here, we demonstrate the existence of a new species of the formerly monotypic genus *Hendersonida*, based on morphology, molecular characters, and phylogenetic information.

The genetic distances observed between the new species and *H. granulata* were 11% for 16S and 16% for COI. These values imply high levels of genetic divergence, even exceeding the mean divergence reported for other munidid species in previous studies (Machordom and Macpherson 2004; Cabezas et al. 2011; Rodríguez-Flores et al. 2019). The phylogenetic tree, however, supported the existence of a common ancestor and a close relationship of *H. granulata* and *H. parvirostris* with respect to other genera, for instance *Paramunida* or *Agononida* (Fig. 1). Moreover, high genetic distances between closely related species of squat lobsters have been already recorded, for instance, in the genus *Phylladiorhynchus* Baba, 1969, where the values can even be higher than 25% for the COI marker (Schnabel and Ahyong 2019). Alternatively, we cannot discard the possibility of a higher rate of extinction in this lineage than in their *Paramunida* relatives, that might account for the scarcity of the taxa and the long branch lengths (Fig. 1). Indeed, given the general values of nucleotide substitution rate for the COI marker, the age of divergence of these two species of *Hendersonida* would be placed by the Early Miocene, at the time when most munidids suffered a notably burst of speciation (Cabezas et al. 2012). Moreover, the granulated surface of the carapace is constant in both species, and seems to be a diagnostic synapomorphy of the genus, in addition to the shape of the sternum, specifically the anterior margin of the sternite 4 (Cabezas and Macpherson 2014).

## Supplementary Material

XML Treatment for Hendersonida parvirostris
